# Preparedness for practice of newly qualified dental professionals in Australia - educator, employer, and consumer perspectives

**DOI:** 10.1186/s12909-022-03476-7

**Published:** 2022-05-23

**Authors:** Rodrigo Mariño, Clare Delany, David Manton, Kate Reid, Julie Satur, Felicity Crombie, Rebecca Wong, Clare McNally, Diego Lopez, Antonio Celentano, Mathew Lim, Michael Morgan

**Affiliations:** 1 Melbourne Dental School, University of Melbourne, Melbourne, Australia; 2 Melbourne Medical School, University of Melbourne, Melbourne, Australia; 3 Centre for Dentistry and Oral Hygiene, UMCG, Groningen, the Netherlands; 4 Faculty of Dentistry, University of Otago, Te Whare Wānanga o Otāgo, Aotearoa, Dunedin, Ōtepoti New Zealand

**Keywords:** Oral health, Oral health professionals, Preparedness to practice, Australia

## Abstract

**Background:**

Limited data regarding the perspectives of other observers (i.e. those who educate, employ or receive care from) of new graduates’ preparedness to practice is available. The present study aimed to explore perceptions of different observers regarding the preparedness to practice and work readiness of newly qualified dental professionals. This broader range of perspectives is crucial to inform the development of educational programs, including continuing professional development, for newly qualified dental professionals, by clarifying the skills, knowledge and behaviours expected by the dental profession and wider public.

**Results:**

Nineteen individual qualitative interviews were undertaken. Interview participants included clinical demonstrators (*n* = 9; 2 Oral Health Therapists; 5 Dentists; and 2 Prosthetists), dental course convenors (*n* = 4), representatives of large employers (*n* = 2), and consumers (*n* = 4). According to this diverse group of respondents, dental students receive adequate theoretical and evidence-based information in their formal learning and teaching activities, which prepares them for practice as dental professionals.

There were no specific clinical areas or procedures where preparedness was highlighted as a major concern. Notwithstanding this, specific graduate skills which would benefit from further training and consolidation were identified, including areas where higher levels of experience would be beneficial. Nonetheless, respondents indicated that new graduates were aware of their limitations and had developed self-discipline and ethics that would allow them to identify conditions/situations where they would not have the experience or expertise to provide care safely.

**Conclusions:**

From an observer perspective, dental students appeared to have gained adequate theoretical and evidence-based information in their formal learning and teaching activities to prepared them to commence practicing safely as dental professionals. Areas were identified in which new graduates were underprepared and when transitional support may be required.

## Introduction

Preparedness to practice for health professionals is defined as having adequate technical skills, clinical knowledge and attributes necessary to practise their chosen profession [1, 2]. This includes the ability to communicate appropriately and interact with the patient and other health staff within their chosen profession [1, 3], supported by protective mechanisms and adaptive skills, community health knowledge and the ability to practice in a culturally safe manner within the context of the ethical and legal expectations of the health care system [4].

In Australia, the Australian Dental Council (ADC) is responsible for accrediting training of dental professionals. The ADC is an independent accreditation authority assigned the accreditation functions for the dental professions by the Dental Board of Australia (DBA) under the National Registration and Accreditation Scheme (NRAS). The ADC works to protect the health and safety of the public by ensuring newly qualified dental professionals have achieved the required professional competencies and meet the standards required of dental professionals in Australia [4].

Additionally, thirteen universities and three registered training organisations have established internal and external review and benchmarking processes and assessments to ensure quality of teaching relevant to the needs of the community with reference to the Australian Qualifications Framework [5]. Such programs aim to produce graduates who meet the ADC’s professional competencies of newly qualified dental professionals [4].

The evaluation of preparedness to practice for health professionals comprises both objective and subjective elements [6]. Curriculum content, opportunities for skills development, and assessment of specific competencies within educational programs provide objective criteria of strengths and weaknesses in dental students’ preparedness for future practice. Subjective elements of preparedness to practice include students’ self-reported confidence and competence, and perceptions of the adequacy of their training. Ensuring that newly qualified dental professionals are adequately prepared to provide safe clinical care also requires that these skills and knowledge are constantly refined through self-directed learning and reflective practice. The ADC publishes competencies for the newly qualified dental professional that cover the areas of professionalism, communication and leadership, critical thinking, health promotion, scientific and clinical knowledge, and patient care [4]. These competencies are developed in conjunction with key informants, including other members of the dental profession and the wider public.

Some authors suggest that perspectives of preparedness to practice should consider both objective criteria related to curriculum content and performance against standards and subjective criteria related to workplace performance [7, 8]. In this regard, employer perspectives may differ from those of graduates because they apply different criteria regarding whether an individual has the knowledge, skills, experience, and attributes suitable for a particular role or within a particular setting.

From a workplace perspective, employers may value certain skills and know-how and they may subjectively rate a new graduates’ preparedness for practice differently to the education provider, the student, or consumers [9]. However, little research has explored the perspectives of different observers of new graduates’ preparedness (i.e., those who educate, employ, or receive care from new graduates) - only one study was found from this perspective, which identified areas of unpreparedness in medical graduates and recommended involving multiple stakeholders when undertaking preparedness research [10]. Such perspectives may align with or be divergent from the self-perceptions of graduates and gaining access to these diverse views and standpoints is likely to advance understanding of dental professionals’ preparedness for practice.

To fill this knowledge gap, and to develop a more comprehensive understanding of dental professionals’ preparedness for practice, the present study aimed to explore a range of perceptions regarding preparedness to practice and work readiness of newly qualified dental professionals. The study aimed to explore the following question: what are the perceptions of preparedness for practice of newly qualified dental professionals of those involved in dental training and education, and from those who employ, or receive care from them? A broader range of perspectives is crucial to inform development of educational programs, including continuing professional development for newly qualified dental professionals, by elucidating the skills, knowledge and behaviours expected by the dental profession and wider public.

## Methods

The study utilised a qualitative research design with semi-structured individual interviews to explore the perspectives of i) those involved in dental training and education (i.e., dental course coordinators; clinical demonstrators); ii) representatives of larger employers in both the public and private sectors; and iii) consumers of newly qualified dental professionals’ preparation for practice. For this study a dental professional is a practitioner in any of registration categories in Australia: dentist; dental hygienist; dental prosthetist; dental therapist or oral health therapist [4].

Approval to conduct the study was obtained from the Department of Medical Education Human Ethics Advisory Group at The University of Melbourne (ethics ID. 1,954,334.3). Recruitment of educators and employers to participate in the study was undertaken through targeted emailing of a research invitation by the ADC to stakeholder groups, with the aim of securing participants across all Australian states and territories, from rural, regional, and metropolitan locations, and in the public and private sector. Potential participants expressed their willingness to undertake an interview by registering their name and email at the end of a quantitative questionnaire designed to gather information from a larger sample [11]. Recruitment was undertaken after the COVID-19 pandemic and associated restrictions commenced: for convenience, recruitment of consumers of dental services was limited to the state of Victoria. For privacy reasons, consumers were approached via a large public oral healthcare provider and asked to indicate their interest in participating in this study to the researchers. Interested participants expressed their willingness to participate directly to the researchers and thus their participation status was not known by the ADC, their employing organisation or health care provider.

Semi-structured individual interviews were conducted by a single interviewer with experience in conducting qualitative interviews (ML) between March and July 2020 at a mutually convenient location face-to-face or by telephone. Each interview lasted an average of 30 minutes and was audiotaped (with participant permission) and transcribed. Interview questions were designed to explore participants’ perceptions with regards to preparedness to practice and work readiness of newly qualified dental professionals. The structure of the interview was developed with reference to the key themes in preparedness for practice for dental professionals identified by systematic literature reviews undertaken by Mahon & Ravindran [12] and our research group as part of a previous quantitative questionnaire study, as well as the results of that questionnaire [11–13]. Two interview guides (one for dental professionals and a modified, less technical version for consumer representatives) were developed by the research team in consultation with a research advisory panel. Tables 1 and 2 include the interview schedules for dental professionals, educators, and employers, and for consumers, respectively.

**Table 1:** Qualitative themes explored with semi-structured interview to educators and employers

1. Self-assessed levels of preparedness for practice by new graduates and the dental profession. 2. Particular areas that you felt new graduates are less prepared for after having completed the survey? If so, which area(s)? 3. Clinical areas that new graduates expressed some uncertainty about managing (i) medical emergencies and (ii) dental trauma. 4. Areas where both the profession and new graduates indicated that they felt new graduates were less prepared: (i) identifying signs of neglect and abuse; and (ii) managing individuals with disabilities or special needs. 5. Concern about how both the profession and new graduates felt about new graduates practicing as health care professionals. In particular, (i) the interface between clinical practice, (ii) patient care and legal obligations; (iii) explaining treatment costs, negotiating fees and the financial pressures of business; and (iv) managing time and stress, balance. 6. Perceived preparedness of new graduates. The graduates themselves.

**Table 2:** Qualitative themes explored with semi-structured interviews to consumers

1. Have you ever been treated by a new graduate oral health professional (dentist, oral health therapist, hygienist, prosthetist)? 2. Are you aware that there are published standards for graduating oral health professionals? 3. Do you think that these reflect what patients expect of new oral health professionals? Do you feel that new oral health professionals are ready to treat patients and enter dental practice? 4. What characteristics or skills do you feel are essential for an ‘oral health professional’ to be ready to practice? 5. Do you have any other comments relating to whether oral health professionals are ready to practice?	

Interviews were transcribed verbatim using Go Transcribe (Go Transcribe: https://go-transcribe.com/) and checked against the initial recordings for accuracy prior to analysis. However no grammatical corrections were made. Initial identification of coding categories was undertaken through systematic reading of the transcribed interviews by members of the research team. Data were stored using NVivo version 12 software (QSR International Pty Ltd., Vic., Australia). Data were analysed using inductive thematic analysis [14] to identify different types of individual perceptions and ideas about preparedness to practice. These ideas were categorised into key themes and the ideas were compared within and across themes. An iterative process of categorising and re-categorising was involved in the thematic analysis. The authors met online regularly over a period of three months of analysis in 2020 to ensure consistency and agreement through the process.

## Results

Nineteen individual qualitative interviews were undertaken. Interview participants included clinical supervisors (*n* = 9; 2 oral health therapists; 5 dentists; and 2 prosthetists), dental course convenors (*n* = 4), representatives of large public employers (*n* = 2), and consumers (*n* = 4). Six participants were from Queensland; four from NSW; two from Western Australia; two from ACT; and five from Victoria. The characteristics of the participants are described in Table 3. Participant characteristics are noted following each quote in the text to identify interviews as educators, clinical supervisors and employers (S), or consumers (CS). Consumers provided comments about their experiences with dental professionals generally, but also specifically their experiences with graduates and student practitioners. Just under half of participants (*n* = 9) were from regional areas including 4 of the clinical supervisors, and 2 each of the course convenors and consumers.

**Table 3:** Characteristics stakeholders interviewed for this study

Interviewee	Sex	State	Location Descriptor	Employment Sector	Dental Qualification
S1	Male	Queensland	Metropolitan	Education	Dentist
S2	Male	Queensland	Regional/Rural	Education	Dentist
S3	Male	NSW^a^	Regional/Rural	Private	Dentist
S4	Female	NSW	Metropolitan	Public/Education	Oral Health Therapist
S5	Female	Queensland	Metropolitan	Public	Oral Health Therapist
S6	Female	Queensland	Metropolitan	Public/Education	Oral Health Therapist
S7	Female	NSW	Regional/Rural	Education	Oral Health Therapist
S8	Male	ACT^a^	Regional/Rural	Private	Prosthetist
S9	Male	WA^a^	Metropolitan	Private	Specialist
S10	Male	Victoria	Metropolitan	Private/Education	Dental Specialist
S11	Male	Queensland	Metropolitan	Education	Specialist
S12	Male	WA	Metropolitan	Private/Education	Dental Specialist
S13	Male	ACT	Regional/Rural	Private / Public	Dental Specialist
S14	Female	NSW	Metropolitan	Private/Education	Dental Specialist
S15	Female	Queensland	Regional/Rural	Education	Dental Therapist
CS1	Male	Victoria	Regional/Rural	Consumer public sector	–
CS2	Male	Victoria	Metropolitan	Consumer Private and public sectors	–
CS3	Male	Victoria	Metropolitan	Consumer public sector	–
CS4	Female	Victoria	Regional/Rural	Consumer public sector	–

^a^*NSW* New South Wales, *WA* Western Australia, *ACT* Australian Capital Territory

Thematic analysis identified seven major themes, with six areas of preparedness for practice (academic and technical competencies, clinical entrepreneurship, communication and interprofessional skills, professional attitude and ethical judgement, protective mechanisms and adaptive skills, and social and community orientation), corresponding to those identified by Mohan and collaborators [12]. In addition to these six areas, a seventh preparedness theme identified from the interviews related to support interventions to increase preparedness. These themes were consistently identified across the different dental schools and professional contexts represented in this study. The following sections describe the key themes.

### Theme 1. Academic and technical competencies

The importance of academic and technical competences was a strong theme to emerge across the interviews. Many of the interviewees commented about the significance of basic training, which was reported to be at the required level to practice safely in Australia. Participants agreed that if graduates had a sound initial knowledge base and motivation, they could consolidate their knowledge once they were immersed in professional practice. Exposure to the clinical environment allows them to keep abreast of new developments in their respective fields and develop as professionals:

“…they are prepared, to a certain degree, with basic knowledge to be able to perform treatments safely. But I do not think it really prepares them until they actually get out there and start doing it 24/7 with those extra pressures on them. Like the communication, or the time management, or the difficult patient that there might be”. (Interviewee S6)

Perceptions varied about the adequacy of clinical exposure during training and graduates’ preparedness to translate knowledge into clinical practice. Particular strengths, such as infection control practice and, especially for Bachelor of Oral Health graduates, oral health promotion were identified.

The consumers expressed a degree of confidence and comfort seeking care from student and graduate dental professionals:

“They seem to know what they're doing…, I'm not impatient. Like I know sometimes that a student might take a bit longer…” (Interviewee CS3)

Interviewees also acknowledged that new graduates had a better grasp of evidence-based knowledge informing practice, than previous cohorts of dental professionals.

“I know when I graduated, I would not have known a very good study from a poor study. You know, now there is a lot more review articles. A lot more, you know, secondary reviews and tertiary review articles”. (Interviewee S3)

Others regarded clinical experience during training as too limited, making new graduates somewhat unprepared for practice and postulated there was insufficient hands-on training throughout the program. Additionally, the need to be selective with patients for teaching purposes was mentioned. There was recognition that it may be difficult to provide a suitable variety of patients and procedures for every student throughout their training, and how this may limit the preparedness of graduates across the full range of practice. New graduates were considered to be “very good with the theory in many ways…” (Interviewee S9), but a lack of practice exposure could affect clinical confidence until they start working in the “real world”. Thus, recognising that clinical experience allows for concepts and theories to be integrated and applied into practice.

Several weaknesses in the preparedness for practice were also identified by participants. These tended to be aspects of practice rarely encountered, making it difficult to provide students with patient experience during their course. Whilst often acknowledging the challenges around frequency and unpredictability of occurrence, participants reported preparedness would be improved by more exposure to:

- Management of medical emergencies: “medical emergencies are hard, […] I just think it’s just an ongoing training thing that everyone needs to have”. (Interviewee S1)
- Dental emergencies and dental trauma:
  “I think they are prepared knowledge-wise, but I don't think they probably feel prepared until they have actually done a trauma victim. Realistically, they are so few and far between. You don't get an avulsed tooth every week walking into a clinic”. (Interviewee S4)
- Identification of neglect and family violence or abuse. Although it was mentioned that current graduates are more prepared than any previous cohort of dental professionals to identify signs of neglect and abuse, there was scope for further development:
  “…lectures on if they suspect domestic violence or child abuse, the mandatory reporting there is a lot of information they get a mandatory reporting”. (Interviewee S14)

Experience in aspects of care, which are the responsibility of training organisations to address were also identified, including:

- Management of referrals and dental emergencies. Regarding dental emergencies, one participant commented that there was limited exposure: “you have one or two sessions a week in the emergency clinic. So, it just… yeah whatever comes in the door. It’s a bit potluck ...” (Interviewee S6)
- Communication skills in difficult situations (including cases of neglect and family violence or abuse). Graduates also require the communication skills to discuss sensitive issues like this and/or have difficult conversations:

“You see the young woman come in black eyes broken front teeth and you had a very good suspicion of what happened and say well what happened? ‘I don't want to talk about it I tripped and fell over’. Where do you go with that?” (Interviewee S14)

- Management of patients with special needs. Participants generally agreed with the need for these populations to be included in the curricula, and were cognisant of the challenge in doing so, particularly in clinical exposure:

“They are probably not getting the exposure that they feel they want, or they need. But again, it's hard because you can't hand-pick patients that come to the clinic to be able to give them that exposure”. (Interviewee S12)

The financial and time pressures placed on both the curricula and institutions to deliver their programs were noted by clinician participants as additional barriers to achieving better academic and skill-based outcomes in these areas.

### Theme 2: clinical entrepreneurship

This theme arose from participants’ discussions about whether and how graduates were prepared to manage the financial considerations of patient care, both from the perspective of business (i.e., generating an income) and confidence with managing patient expectations of the cost of dental care. Consumers also indicated that discussions about cost were important to them when seeking dental care.

Participants typically began with broad judgements about graduate approaches to the financial aspects of dental practice. The participants identified that those graduates from programs in the Vocational Education and Training sector received training that was consistent with the respective training packages. For those graduating from a university program, the level and amount of learning about the business aspects of practice was variable, but there was a general sense of lack of preparedness. For some, this was due to the nature of the clinical exposure and patient base (i.e., public sector):

“They come out of university never having to speak to patients about costs and money and for a lot of new graduates… it is also very awkward to talk to patients about money and about costs and dealing with reactions of people who might find the costs quite shocking”. (Interviewee S9)

Nevertheless, approaches to addressing these concerns were seldom raised. Some questioned the role of dental schools in preparing dental graduates for the entrepreneurial aspects of the profession.

Solutions for these concerns were seldom raised, and others questioned the role of dental schools in preparing graduates for the entrepreneurial aspects of the profession.

“…I think there are other things that they should be learning or spending more time on rather than how to speak to patients about charging fees. So, I would be very firmly in the camp of ‘okay it's an issue, but the university can't teach the students everything that they need to know when they graduate’”. (Interviewee S10)

There were also some comments about how clinical training experiences in the private sector may be able to help provide exposure to the financial aspects of patient care. It was also noted that some dental schools were able to provide this experience and exposure. There was recognition of experiences and exposure to these aspects in some dental schools. On the other hand, educators and employers commented that students advocated some entrepreneurial and commercial aspects, but for the benefit of the patients.

There was also concern regarding the challenges related to the high cost of becoming a dental professional in Australia, and how this may influence patient care decisions and discussions.

“I do worry that there is a lack of professionalism coming out with the new graduates from the point of view that they are under so much pressure with the commercialism”. (Interviewee S8)

The differences between private and public practice were also contrasted by several participants, with the differing financial drivers in these settings highlighted (i.e., a set salary vs. a commission). Also, the perception of private versus public practice, and how these modes of practice are presented to students/graduates. Some participants expressed some concerns about the impact of these issues on graduate dental professionals:

“… and often from the graduates coming out, the public sector is its second-best choice. You know, if you cannot get a job in private practice, you will have to work in the public sector”. (Interviewee S8)

Numerous concerns were expressed about the potential threats of emphasising entrepreneurial aspects of the profession, and increased exposure to private practice, which traditionally reward more surgical and aggressive treatments over prevention and long-term patient health outcomes:

“But it is still driven by the drill, fill and bill mentality, and the attitude of many of the students remains that. And the behaviour of people on graduation remains that”. (Interviewee S11)

### Theme 3: communication and interprofessional skills

This theme captured participants’ discussion about how communication and capacity to work within interprofessional teams is a key element in bridging the division between the clinicians and patients across ages and socio-economic differences. Most participants indicated that the communication skills of new graduates were acceptable for practice. Employers reported that new graduates communicated effectively with their patients, guardians or carers and that communication skills were not a particularly significant concern for them.

A strong theme that emerged was that better communication skills could help new graduates develop a better rapport with patients and have more success with treatment. It was also appreciated that new graduates might be better at, and feel more comfortable with asking sensitive questions than previous cohorts of dental professionals:

“I know that that they are pretty okay with asking patients at the hospital about cannabis drug use, even some 15-year-old in pain. They have said: any possibility you could be pregnant?; any recreational drugs?”. (Interviewee S7)

Despite this, communication was identified by participants as one aspect of graduate practice that was challenging. Specific groups in the community were highlighted as possible areas of concern in relation to communication. For example, culturally and linguistically diverse groups were perceived as a communication challenge for new graduates. Whilst language might not be a problem for basic communication, communication difficulties might also occur because of differing cultural meanings or interpretations, thereby limiting effective communication [15]:

“… [when English is not their first language] students particularly struggle in my experience. I do not think they get enough training in it”. (Interviewee S15)

Some participants indicated that some programs appeared to be addressing these communication issues, albeit based on the experiences of academics undertaking other (i.e., clinical governance) roles:

“… And it goes throughout the entire program and there are specific assessment tasks that are linked to communication skills. And we also started investing to make our students do certain activities that are really directed at communication”. (Interviewee S7)

Participants also provided some discussion of the context for communication as being an influence on the graduates’ perception of private versus public practices:

“What they might struggle with is actually dealing with more demanding patients I suppose the ones that are, … if they go into private practice, paying for their treatment …” (Interviewee S4)

Communication issues are common complaints across all health professions [16]. For some participants there were clear benefits of good communication skills in terms of “work-readiness” in preventing complaints:

“… 90% of those complaints were about lack of communication and the patient did not understand what was being told, that they get told to sign off on this paper and they had no idea”. (Interviewee S14)

Regarding the legal scenario, it was particularly mentioned as being much more complex. In fact, participants appreciated that the current working scenario was far more complex, and with additional legal demands in the contemporary environment compared to previous experiences. Apart from notifications against dental professionals relating to clinical care issues, other main issues for dental professionals were billing, communication, and informed consent, all of which relate to communication [17].

The relationship between empathic care and communication was also addressed by several participants; however, it was not a particularly strong theme. This consideration of empathy included both the teaching and learning of empathic skills and attitudes:

“… sometimes with some people you cannot teach ethics and you cannot teach empathy. We certainly try to feed all of that in and then let them know that the regulators are there to protect the public”. (Interviewee S4)

Communication was an area of practice raised by the consumers. In one consumer’s experience with dental students, they appeared to appreciate that describing the treatment plan without using dental jargon could be challenging:

“And they explained everything as good as they could, which really is a little pointless. As you know, they are talking about the number of the teeth, what they got to do or inclusions or that sort of stuff, they tell you, but most people wouldn't have a clue what they're talking about.” (Interviewee CS1)

### Theme 4: professional attitudes and ethical judgment

This theme represents participants’ discussion about the type of ethical framework and knowledge important for professional practice. There was strong agreement that new graduates displayed a professional attitude to their work and made ethical judgements in patient care and professional decision making. Participants were generally satisfied with these aspects of new graduates’ practice where they noted that new graduates adopted a more conservative approach to practice.

Some participants focused on this theme with respect to the training provider and believed that there was an opportunity to raise awareness of the regulatory schemes governing dental practice:

“I am completely paranoid that our students will leave and do something wrong. [...] I actually find that a lot of practitioners actually don't know what the role of the regulator is… and that is a little scary”. (Interviewee S4)

Others believed that awareness of national standards and other health system-based issues was important because it may influence graduates’ perceptions of risk:

“…but just being aware of the national standards and that, you know, hospital health service districts have to go through accreditation and meet the national standards now”. (Interviewee S1)

Consideration was also given to the legal aspects of dental practice:

“They do have a knowledge of the basics of the legality of what they are practicing and things around that, and that's a dynamic situation that changes a lot”. (Interviewee S13)

Professional attitudes, including commitment to patient centred care principles such as facilitating continuity and coordination of care between dental and non-dental practitioners were considered by participants:. One participant was particularly enthusiastic on the role of multidisciplinary healthcare in the management of dental issues:

“…it really has to be part of uniform seamless clinical handover between whoever and that patient as they get dentally fit…” (Interviewee S1)

One participant was particularly enthusiastic in their desire for greater integration with other health professionals to optimise patient care.

Participants indicated that new graduates were also exposed to commercial pressures, which might compromise their ability to practise ethically. Although graduates recognised their ethical responsibilities, they were challenged to navigate these tensions in the context of their responsibility to an employer:

“I know several young graduates come and talk to me and say … ‘I did this, and I did this, and I did that... and I should not have really, I know that. And you know… my boss is asking me to do this…” (Interviewee S9)

Some participants expressed concerns about how dentistry is practiced currently, particularly, a perceived decline in professional values related to the commercialisation of health care systems in which the primary concern for investors was the maximisation of income [18].

“I do worry that there is a lack of professionalism coming out with the new graduates from the point of view that they are under so much pressure with the commercialism.” (Interviewee S8)

### Theme 5: protective mechanisms are occupational health risks

Participants’ views of the skills and attributes displayed by graduate dental professionals to manage their own health and well-being as a new graduate were explored in this theme.

For some participants, reflection also appeared to overlap with resilience, indicating that if they are not resilient - “Practice is going to be tough…” (Interviewee S15), but this was not consistent across all interviewees. It was also acknowledged that, while extremely important, the ability of some graduates to reflect on and/or evaluate their own performance might be challenging:

“…new graduates who are overestimating their skill levels. […] and they're the ones that will be the ones that will get themselves into strife because they're going to take on the cases that they probably shouldn't”. (Interviewee S4)

Reflection and encouragement to undertake Continuing Professional Development (CPD) and self-directed learning were also considered a protective mechanism within the professional training of dental professionals as a protective mechanism. In general, participants were positive about the self-reflection skills of new graduates and the personal qualities that: “…comes naturally with the type of people that apply for the course”. (Interviewee S2).

Related to protective mechanisms are occupational health risks. In dentistry, as in all professions, occupational health risks are a reality, most often impacting hearing, musculoskeletal function, and psychological equilibrium [19]. Consistent with this concern, consideration, albeit limited, was also given by participants to the impacts of practice on a practitioner’s musculoskeletal health: “…dentistry is a hard profession. It is taxing on your back. Your neck, musculoskeletal”. (Interviewee S1).

Consumers also indicated some areas of concern about dental professionals’ own health and expressed concerns that these aspects should be better covered during their training as professionals (e.g., students’ well-being).

### Theme 6: social and community orientation

Participant perceptions of dental professional graduates’ preparedness with respect to knowledge and ability to provide culturally safe care for patients from a variety of culturally and linguistically diverse backgrounds, and Aboriginal and/or Torres Strait Islander peoples, were explored in this theme. All participants recognised the need for new graduates to be prepared to manage patients from a diverse variety of backgrounds. None of the participants identified significant concerns about new graduates’ preparedness to provide appropriate care for Aboriginal and/or Torres Strait Islander peoples, or patients from CALD backgrounds. They perceived that new graduates have undertaken a degree of training around cultural competence and safety, and awareness through conventional and online courses. However, participants also suggested that a true appreciation or application of this awareness was only able to be gained from their own learning, through firsthand exposure and practice through clinical placements and exposure:

“… I don't think until they get out into the real world - and I'm speaking more in Aboriginal remote communities or island places”. (Interviewee S4)

Participants also commented on the preparedness of graduates to care for patients with special needs. Several participants identified relationships with organisations where exposure to special care populations was available (e.g., mental health, adults with disabilities). They generally agreed with the need for these populations to be included in the curricula, and were cognisant of the challenge in doing so, particularly in clinical exposure:

“They are probably not getting the exposure that they feel they want, or they need. But again, it's hard because you can't hand-pick patients that come to the clinic to be able to give them that exposure”. (Interviewee S12)

Participants also commented on the preparedness of graduates to manage clinical scenarios with a family violence or abuse component. There was recognition of the fact that graduates were exposed to this area through the curricula:

“…now they have had lectures on if they suspect domestic violence or child abuse, the mandatory reporting. There is a lot of information they get [on] mandatory reporting”. (Interviewee S4)

However, participants suggested that more could be done regarding domestic violence:

“I think a lot of General Practitioners [*who are*] experienced folks aren't particularly aware of it either. It's something our [*dental*] profession could do with more education”. (Interviewee S15)

### Theme 7: interventions to increase preparedness

Participants provided several recommendations of strategies to optimise opportunities for new graduates’ preparedness for practice including more structured or additional supports, such as internships, after graduation mentoring, and extended or additional clinical placements during initial training. They acknowledged that new graduates were highly motivated and, as representatives of the future workforce, should be highly supported. Mentoring was identified consistently as one aspect that could be beneficial for newly graduated dental professionals. Participants described formal mentoring as beneficial for everybody, but particularly for recent graduates, to support mental health and well-being:

“Mentoring from a work point of view, but also from a spiritual point of view that are trying to help them get organized, not to get upset about things that go wrong through the day, but they really need someone helping them to learn the case and treatment planning I guess”. (Interviewee S1)

Participants also suggested that the role undertaken initially by clinical tutors and educators during training could be continued in the employment context and throughout graduates’ professional life.

Preparedness for practice was viewed through an ‘exposure’ lens by some participants. As described previously, exposure to private practice was mentioned, however, it was acknowledged that in Australia, private practice does not offer the same level of mentoring programs for new graduates as the public sector. Also ‘exposure’ to rural practice, ‘even minimal’, was identified as being another element needed in pre-professional dental curricula.

Consumers also reflected on the training received by students and graduate health professionals and areas that could be improved. Consumers were aware of the pace of new knowledge changes and were interested in dental professionals’ ability to maintain their skills and knowledge. Consumers believed that training to be a dental professional should include aspects related to the mental health of patients and self-care of the professionals themselves. Consumers also highlighted the importance of new graduates’ ability to work in a professional and safe way with a variety of populations and members of the community, including people from different cultural backgrounds, low socioeconomic backgrounds, those with mental illness or a cognitive impairment.

## Discussion

This research explored the perceptions of a range of observers about the preparedness for dental practice of Australian newly graduated dental professionals. Participants in this study were diverse in terms of dental professions, and location. The preparedness themes identified in this study corresponded with those identified by Mohan et al. [12] (i.e., academic and technical competencies, clinical entrepreneurship, communication and interprofessional skills, professional attitude and ethical judgement, protective mechanisms and adaptive skills, and social and community orientation) with an additional theme (interventions to support preparedness) that was unique to this study.

Australian dental schools have different curricula, different approaches to teaching and learning, and different admission processes. However, findings from this study suggest that there are no significant differences or trends regarding preparedness for practice between graduates from the different schools represented.

Preparedness for practice indicates that newly graduated dental professionals enter the health care system with acceptable levels of clinical skill and competencies, and the research identified no specific clinical areas or procedures as insufficient. However, being prepared for practice in these ways, does not imply competence [12]. Educators and employers acknowledged that dental training received at dental schools was not an end in itself, and that further clinical experience is required to refine the skills required to work with patients and colleagues. Consistent with the literature, it was generally acknowledged that consolidating competencies in clinical practice is a lifelong learning process which can only be achieved with practice experience [12, 20].

There were some specific gaps in graduate skills identified in the research. These were areas where graduates could benefit from further training and consolidation, as well as areas where higher levels of experience might be required. Employer participants in particular, expressed the view that new graduates do not get exposure to all types of clinical experiences. These included, among others, management of referrals, management of patients with special needs, communication skills in difficult situations. For example, good communication skills and empathy/compassion help to foster a good practitioner-patient relationship, regardless of the patient’s ethnic or cultural background, and these professional characteristics are particularly important when helping patients who might remain attached to their original values and health practices [21]. In addition, culturally and linguistically diverse groups were perceived as a communication challenge for new graduates. Whilst language might not be a problem for basic communication, communication difficulties might also occur because of differing cultural meanings or interpretations, thereby limiting effective communication [15]. As Australia is a culturally and linguistically diverse (CALD) country, the need to be proficient in working in this context was highlighted. Within this socio-cultural context, the Australian dental workforce must not only be clinically competent, but also have the ability to communicate effectively with diverse patients and be culturally safe in providing treatment, including throughout the entire consultation as an essential part of the dental practice [22, 23].

In this respect, some participants [i.e., employers) suggested that the new graduates were far better prepared to address cultural aspects of care than they themselves had been with respect to the psychosocial aspects. Other participants suggested that new graduates were not necessarily well prepared to ‘look outside of the mouth’ or look at the patient holistically. This aspect of health professional training appeared to be either lacking in dental training programs or given scant attention at one or two points through a training program. It appears that whilst providers are improving teaching to ensure graduates can meet the needs of culturally and linguistically diverse populations, and of Aboriginal and Torres Strait Islander peoples, there is an opportunity to strengthen the theoretical and clinical teaching and engagement with psychosocial aspects of care.

One of the key findings of the study was that graduates might be less prepared for treating emergencies, both medical and dental. Several other studies [10, 24, 25] have similarly found that new graduates lack these types of skills or experience. Participants acknowledged that the focus of training students in dental schools, was mostly on equipping students with a set of basic skills for starting clinical oral health care work, and that there were logistical challenges to include “real-world” experience within clinical training environments. One implication of this finding is to consider national benchmarking for topics such as medical and dental emergencies because of the difficulties in ensuring equitable experience for students to acquire these skills.

A strong theme in this research was an acknowledgement by participants that new graduates were aware of their limitations with respect to dental practice. They highlighted graduates’ self-discipline and ethical framework that assisted graduates to acknowledge their own limitations and identify those conditions/situations where they might not have the experience or expertise to provide care safely.

The perceptions of students lacking sufficient preparedness to practice from supervisors and employers was related to the entrepreneurial, financial, and administrative aspects of dental practice. There is an expectation that new graduates must adjust to business activity when they start practicing, particularly, in the private sector, such as discussing and charging fees with patients. However, these types of skills are difficult to formally teach and are best learned on the job [23]. Thus, there is a limit to the extent to which certain aspects of work can be learned in a classroom setting, simulation, or even in a clinical environment and then transferred to a real-life clinical setting [2]. Additionally, this is an aspect more related to employability, in particular in the private health care sector [12].

The data collection for this research occurred during the COVID-19 pandemic. The pandemic stimulated the use of telehealth, and it now seems that this modality will remain [26]. Nonetheless, the use of Information and Communication Technology (ICT) and Digital Health as a competency was not mentioned at all by the participants. However, these and other developments of ICT, such as with artificial intelligence, robotics, self-learning machines or the need to analyse large amounts of data, will require the development of new competencies among health professionals [27]. Thus, preparedness to practice may evolve and change as well. According to Fejerskov and collaborators [28], dental professionals should also be able to communicate with developers, designers, linguists, programmers, engineers, or psychologists, with the expertise to understand human behaviours and sociological phenomena to meet the health demands of their community and provide advice to people to adopt healthy behaviours and avoid unhealthy ones.

Preparedness for practice means that a graduate is ready to practice independently in a diverse range of environments, not only being competent for clinical practice [12]. New graduates may not be fully prepared for the business aspect of private dental practice; however, according to this research, there were few concerns about the clinical skills of graduates. Furthermore, a dental professional is a professional who is trained to make multiple therapeutic decisions and recommendations that affect the lives of their patients, with professionalism and with a high degree of critical thinking, whether in private practice or in community clinics, hospitals, or other public sector establishments. There are also other career paths for dental professionals (e.g., educators, researchers, industry) [29] which were not mentioned by stakeholders.

As in any research, this study was not without limitations. For example, the invitation to participate was sent in late February 2020, just prior to the COVID-19 pandemic lockdown in many parts of Australia, which may have affected recruitment of participants. Another limitation was the self-reported nature of the responses, that may have either overstated or understated preparedness for practice assessments. Despite these limitations, the present study achieved representation in terms of dental professions and dental schools, suggesting that the present findings contribute significantly to current knowledge and are transferable to other jurisdictions with some degree of confidence.

This study explored the perceptions of a range of informants about the preparedness for dental practice of Australian newly graduated dental professionals. According to participants, dental students appear to be acquiring adequate theoretical and evidence-based information in their formal learning and teaching activities, which adequately prepares them to begin practicing as dental professionals. The study identified some areas in which new graduates are underprepared and when transitional support may be required.

It is hoped that these findings provide dental educators and curriculum developers with an overview of how different participants view the preparedness of dental graduates in the current Australian context. The study expands understanding about preparedness beyond graduate based research to include the lesser-known perspective of those who educate, employ, and receive care from new graduate clinicians. Graduates can only assess preparedness to practice in view of their own experience [10], and this might contribute to inappropriate expectations about performance [3]. The present study may also highlight interventions which can be implemented to support newly graduated dental professionals in their transition from student to clinician.

## Data Availability

The datasets generated and/or analysed during the current study are not publicly available due to the ethics approval granted on the basis that only researchers involved in the study can access the de-identified data. The minimum retention period is five years from publication. Supporting documents are available upon request to the corresponding author.

## Abbreviations

ADC: Australian Dental Council
AHPRA: Australian Health Practitioner Regulation Agency
CALD: Culturally and Linguistically Diverse
CPD: Continuous Professional Development
ICT: Information and Communication Technology
NRAS: National Registration and Accreditation Scheme
